# Introduction to Biomaterials in Innate Immunity

**DOI:** 10.1039/d4ma90056a

**Published:** 2024-05-17

**Authors:** Erika Moore, Shreya A. Raghavan

**Affiliations:** a Fischell Department of Bioengineering, University of Maryland College Park USA emt@umd.edu; b Department of Biomedical Engineering, Texas A&M University USA sraghavan@tamu.edu; c Regional Excellence Center in Cancer, Texas A&M University USA

## Abstract

Erika Moore and Shreya A Raghavan introduce the *Journal of Materials Chemistry B* and *Materials Advances* joint themed issue on Biomaterials in Innate Immunity.
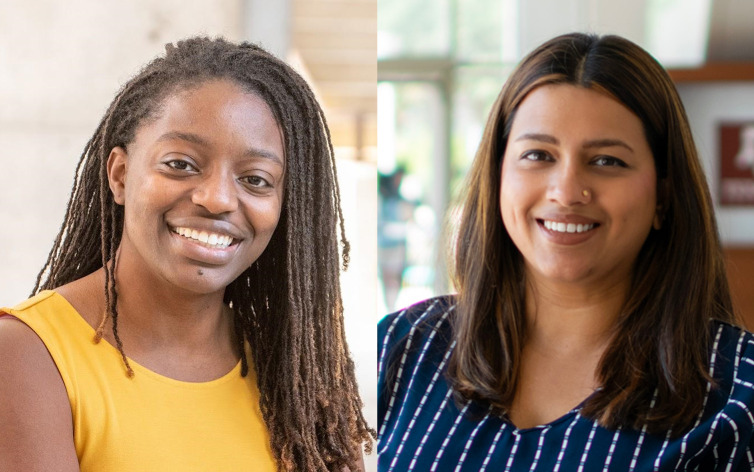

## Biomaterials in innate immunity: a knowledge-driven approach to immune-modulation

We are delighted to present this comprehensive issue on “Biomaterials in Innate Immunity”. Modulation of innate immunity is an attractive therapeutic target, be it to improve outcomes in pathological contexts like cancer or boosting tissue regeneration following injury and inflammation. Wide-spread adoption of innate immunomodulation, however, requires the intersection of biomaterials engineering and immunobiology.

The collection of articles in this themed issue focus on how biomaterials can be harnessed to decode and modulate the behavior of innate immune cells in the contexts of regeneration and disease. In our selection of articles, specific attention is drawn to biomaterial design and formulations, while integrating newer spectroscopic and bioinformatics techniques. These diverse biomaterials strategies ultimately converge into a knowledge-driven approach to modulate innate immune activity.

First, Galindo *et al.* review various biomaterial formulations to modulate inflammation and promote functional tissue repair within the central nervous system (https://doi.org/10.1039/D3MA00736G). The article highlights the versatility of biomaterials, delivered as nanoparticles, hydrogels, implantable scaffolds or even used as neural probe coatings. The ultimate goal is to use biomaterials to control local neuro-inflammation and promote axonal elongation and mitigate glial scarring. Complementing this notion of local control, Sosnik *et al.* demonstrate that a drug-free polymer-polysaccharide nanoparticle can actively reprogram innate immune cells like macrophages engaged in inflammation (https://doi.org/10.1039/D3TB01397A). Combining *in vitro* approaches and bioinformatics, they demonstrate that the nanoparticle biomaterial has the potential to actively reprogram macrophages *via* local delivery producing local anti-inflammatory effects. This work underscores the intricate relationship between material structure informing cell function which could pave the way for their application in the therapy of different inflammatory conditions, especially by local delivery.

Evers *et al.* lay the groundwork for how cellular mechanics of macrophages are altered as they traverse through different environments in the body from bone marrow through circulation to inflamed tissues like tumors (https://doi.org/10.1039/D3MA01107K). The article presents single-cell mechanical characterization of tumor-associated macrophages and offers insight into underlying mechanical regulation in tumor-associated macrophages. Building off this theme that materials can be used to guide biophysical responses as well, Davis *et al.* review the importance of structural design in delivering immunomodulatory cargo to innate immune cells for cancer therapy (https://doi.org/10.1039/D3TB01677C). The article highlights the potential of biomaterials in enhancing therapeutic outcomes through targeted design strategies.

Complementing this theme strongly, Boboltz *et al.* engineer a synthetic immune-inspired biomaterial based on neutrophil extracellular traps (NETs) combined with viscoelastic mucin hydrogels (https://doi.org/10.1039/D3TB01489D). With careful characterization of mechanical properties of their synthetic immune inspired hydrogel, the work investigates immunity-mediated airway dysfunction in cystic fibrosis.

## Conclusion

This issue serves as a comprehensive amalgam of cutting-edge research at the intersection of biomaterials and innate immunity. By elucidating the mechanisms underlying biomaterial–immune cell interactions and exploring their therapeutic implications across diverse disease contexts, these articles pave the way for transformative advancements in leveraging the innate immune system for therapeutic impact. We hope that this collection inspires further exploration, collaboration, and innovation, ultimately leading to improved diagnostics, therapeutics, and patient outcomes.

## Supplementary Material

